# Autism as an adaptive common variant pathway for human brain development

**DOI:** 10.1016/j.dcn.2017.02.004

**Published:** 2017-02-09

**Authors:** Mark H. Johnson

**Affiliations:** Centre for Brain and Cognitive Development, Department of Psychological Science, Birkbeck, University of London, Henry Wellcome Building, Malet Street, London WC1E 7HX, United Kingdom

## Abstract

While research on focal perinatal lesions has provided evidence for recovery of function, much less is known about processes of brain adaptation resulting from mild but widespread disturbances to neural processing over the early years (such as alterations in synaptic efficiency). Rather than being viewed as a direct behavioral consequence of life-long neural dysfunction, I propose that autism is best viewed as the end result of engaging adaptive processes during a sensitive period. From this perspective, autism is not appropriately described as a disorder of neurodevelopment, but rather as an adaptive common variant pathway of human functional brain development.

## 1 Introduction

A variety of different pre and perinatal factors can lead to diffuse and widespread atypicalities in neural processing during the first years of life. The consequences of such events for later development are much less well understood then are the effects of more punctate and focal damage, such as those arising from discrete perinatal neurovascular events. In the latter case, substantive evidence supports the triggering of adaptive and compensatory processes within remaining intact tissue that help restore, to the extent possible, the typical trajectory of postnatal human brain development. However, much less consideration has been given to processes of adaptation engaged following diffuse and widespread differences in the fidelity of signal processing, or in the homeostatic neurochemistry related to the synapse. In this paper I further develop the idea that some behaviourally-defined clinical phenotypes, such as autism, are the developmental consequence of natural chain of adaptive responses to such atypicalities in early life neural processing.

A variety of homeostatic processes in the brain ensure optimal balances in key factors such as excitation/inhibition (E/I) balance, and neurotransmitter balance (Turrigiano, 2011). In contrast to some well-studied local cellular and molecular homeostatic mechanisms that can restore local adaptive balance, we know considerably less about the whole brain and neural systems level adaptive processes, and even less about their compensatory responses in the face of altered signal processing at the synapse. This gap in our knowledge may be critical given that common developmental disorders, such as autism and ADHD, are known to result from both intrinsic and environmental factors; in other words, the way a particular brain adapts during ontogeny to its individual social and physical environment. Furthermore, the way an environment is sampled and perceived early in life itself depends on the specific properties of the brain processing it. Thus, the “effective environment” experienced by some infants may be different simply due to their own particular neural processing limitations.

In a recent paper we speculated that the diagnostic behavioral symptoms of autism are the result of processes of early life adaptation in response to atypical neural signal processing, potentially at the synapse (Johnson et al., 2015). This sub-optimal quality signal processing may be caused by genetic or environmental effects, sensory limitations, or most often by combinations of factors. Nevertheless, the adaptive response of the developmental trajectory of the human brain will be similar, and the end result of this trajectory, we argued, is the autism diagnostic behavioral phenotype. From this perspective, the coherence of the clinical syndrome originates in the brain’s unitary response to different kinds of altered synaptic processing, possibly reflected in signal-to-noise ratio. We proposed that a series of compensatory and adaptive processes trigger an alternative trajectory of subsequent development, resulting in the majority of the behavioral phenotype associated with an autism diagnosis. By analogy, a single systemic adaptive response such as elevated body temperature (fever) can be triggered by many different causal factors (bacterial, viral, etc.). According to this view, autism should not be described as a *disorder* of neurodevelopment *;* but rather as a perfectly ordered developmental response in the face of an unusual starting state.

In this paper I address some key issues raised by this new perspective. First, if general factors drive the neurodevelopmental pathway to autism, how does the apparent domain-specificity of the cognitive and behavioral profile of the syndrome arise (Question 1)? Second, what are the mechanisms that underlie the adjustments in whole brain systems to accommodate early differences in synaptic processing (Question 2)? Third, why is it that differences in the functioning of specific brain regions are associated with autism (Question 3)? And finally, to what extent is there a sensitive period early in life within which the adaptive changes must occur (Question 4)? I will conclude by discussing some future directions derived from the new perspective. I begin, however, by making the case for considering whole brain adaptation in human development.

## 2 Whole brain adaptation

Processes in the brain can be observed at multiple levels of organization from molecular to cellular to large-scale systems. Within neuroscience, processes of adaptation have generally been studied at molecular and cellular levels, with less focus on how large-scale neural pathways and systems can compensate for either focal or diffuse disturbances. Two reasons for focusing on this ‘whole brain’ level of description of the nervous system are: (1) to fully understand processes of ontogenetic adaptation we need to consider the whole brain, where evidence shows that distant neural systems and regions can adjust to compensate for poor functioning or damage elsewhere, and (2) common developmental disorders are associated with widespread changes in the functioning of large-scale neural networks, even though these often have multiple different underlying molecular and cellular correlates (Johnson, 2015). Thus, in linking the brain to clinical diagnostic behaviors in developmental psychopathology we need to bridge our understanding with models of whole brain systems function and dysfunction.

In parallel, while the concept of adaptation has had multiple definitions within developmental cognitive neuroscience, these have generally been taken to refer to neural processes or behaviors that are shaped by recurrent problems that faced ancestral populations (e.g., see Bjorklund, 2015). My use of the term is as applied to the restoration of a homeostatic balance after a perturbation in individual development; in other words, *ontogenetic* rather than *phylogenetic* adaptation.

It is important to note that ontogenetic brain adaptation does not necessarily lead to a typical outcome. In child psychiatry the concept of r *esilience* is commonly used to refer to the extent to which an individual withstands or recovers from early disturbance of their developmental trajectory to achieve normality (Cicchetti, 2013, Cicchetti and Curtis, 2007, Masten, 2007). However, *ontogentic adaptation* refers to a broader class of processes in which a given individual’s brain maximizes its fit to the environment in ways that may, or may not, result in a neurotypical behavioral phenotype. Computational models of the cerebral cortex suggest that a range of different starting state disturbances result in one of a small number of atypical outcome phenotypes; a many-to-few mapping (see Oliver et al., 2000).

As stated earlier, we have previously presented the hypothesis that poor quality signal processing early in life, mediated through synaptic contacts, leads to the adaptive developmental trajectory that results in autism (Johnson et al., 2015). We hypothesized that this adaptive trajectory was the product of four types of whole brain adaptation that also drive the typical developmental trajectory. First, *redundancy* – the existence of duplicated functions or neural systems that can compensate for the loss of another under most circumstances. Second, *reorganization* – the reallocation of functions to regions or networks as orchestrated by critical hubs. Third, changes in the *timing of developmental trajectories* to compensate for poor sampling of information from the early environment. Fourth, *niche construction* – the process by which individuals select and construct an environment that best suits their own individual brain’s processing style. With regard to the latter we argued that differences in attentional style observed in some developmental disorders reflect the adaptive strategies of the brain given the limitations and capacities of its processing. Since impaired synaptic processing will mean sparser and less reliable sampling of information from the environment, the focal attentional style characteristic of autism restricts the quantity of information flow to make it more manageable (Johnson et al., 2015). Thus, an overly focal style of attention (characteristic of autism) could reflect parallel processing limits that mean it is beneficial to restrict sensory input to a single channel or area of external space. A second example we previously discussed is the selection for processing features of the environment that are more temporally predictable (and that could thus better suit a brain with poor quality signal processing). Learnability is therefore shifted towards simpler structures, which often tend to be repetitive, mechanical, self-controlled stimulation (such as those generated in repetitive behaviors), to the detriment of processing the more dynamic and variable information associated with typical social interaction, and a gradual withdrawal from the social world. Focusing attention and processing on the more predictable physical world and withdrawing from the less predictable and multisensory social world may thus represent an coherent adaptive response of the brain to difficulties in parallel processing of more dynamic and varying sensory inputs. In other words, the “effective environment” experienced by a developing brain will partly result selection of a sensory environment that best suits its own processing capacities.

## 3 Causes and consequences of diffuse brain damage around birth

While there is an extensive literature on compensatory processes of the brain following acute brain damage during the perinatal period, as stated above much less is known about the adaptive processes triggered by mild and diffuse atypicalities in neural or sensory processing over the early years. The literature on the effects of acquired pre and perinatal localized brain damage on subsequent development has in some cases shown the brain’s remarkable ability to reorganize in a compensatory manner to achieve near typical outcomes; neural “resilience”. For example, large-scale prospective studies of language in children who suffered a single focal unilateral injury event to either the right or left hemisphere before six months of age show some degree of general developmental delay in measures of language (such as lexical, grammatical and discourse structure) regardless of lesion site (Stiles et al., 2002). However, importantly, these disadvantages appeared to resolve over time in the children with focal lesions, allowing them to score within the typical range. Subsequently, delays can re-appear at the next steps of development in language acquisition, a pattern consistent with functional recovery to typical performance (resilience) being a reoccurring event during key points of development (Reilly et al., 1998). It is important to note that this example is merely illustrative of a large literature, and there are differences in findings depending on lesion site and domain of cognition. Nevertheless, where these reorganizations of brain function to achieve near-typical outcomes occur they are probably attributable to intact regions with typical microstructure and neurochemistry being able to re-configure functional connectivity in such a way as to support the necessary computations, illustrating a potential many-to-one structure-function mapping at early stages of development (Park and Friston, 2013).

While in some cases focal perinatal brain damage can elicit resilience as above, discrete ischemic events can also have secondary and more widespread consequences for brain function (Volpe, 2009). Some of these secondary effects may be part of the local adaptive response, while others may be toxic. For example, following Periventricular Leukomalacia (a white matter brain injury that effects newborns) primary focal events involve cellular damage and glial “scars”, while secondary more diffuse effects occur in white matter such as increases in astrocytes and microglia, and an initial decrease in oligodendrocytes that can lead to hypo-myelination. Animal models show that these secondary effects can result in diffuse signal processing abnormalities (see Volpe, 2009 for review). In cases where secondary effects are sufficiently widespread, the usual options for alternative structure-function mappings in the brain may be disrupted, making the typical developmental trajectory less attainable.

Several authors have suggested graded scales of the extent of neonatal brain injury with the purpose of better predicting later outcome (e.g., Low et al., 1988, Marlow et al., 2005, Van Handel et al., 2007). For example, a common secondary consequence of perinatal asphyxia was neonatal encephalopathy (NE), a clinical syndrome of disturbed neurological function sometimes accompanied by seizures. While now commonly treated by head cooling (Edwards, 2009), NE has been typically graded on one of several severity scores as being mild, moderate or severe (Van Handel et al., 2007). While these are inevitably coarse categories for complex and diffuse brain disturbances, they nevertheless give us the opportunity to assess the consequences of different degrees of early perturbation to the developing brain. Mild NE rarely leads to significant later intellectual or cognitive problems, demonstrating the resilience of the neonatal brain in the face of brief (less than 24 h) adverse events, similar to the focal cortical lesions described above. In contrast, severe NE usually results in significant developmental delay, low IQ, and poor educational attainment (reviewed in Van Handel et al., 2007). Most relevant for the present discussion, moderate NE had more variable outcomes. While these children often scored within development norms from infancy to school years, some individuals had deficits in receptive vocabulary, language and visuo-motor integration, in addition to raised rates of hyperactivity and autism (Badawi et al., 2006).

A systematic review of the pre and perinatal factors associated with later autism reveals one of the most significant factors across studies is intrapartum hypoxia, and its related measures such as low Apgar score (Kolevzon et al., 2007). Hypoxic effects are usually widespread in the brain, albeit that the hippocampus and thalamus may be particularly susceptible. It is thus conceivable that intrapartum hypoxic injury to the brain is often of the mild and diffuse kind that subsequently triggers the alternative developmental trajectory leading to the behavioral phenotype of autism.

These examples illustrate a general principle of developmental pathways (Waddington, 1966) that the typical route (chreod) is generally well buffered against minor or transient perturbations (mild NE), but that more significant and longer lasting disruption within a sensitive period can divert development to an alternate pathway in which a different profile of abilities, disabilities and behaviors can emerge (moderate NE). Finally, a more severe and long lasting disruption of development will exceed the limits of adaptation resulting in slow progression down any developmental pathway, and poor life-long outcomes over all domains (severe NE).

We now turn to the specific questions raised earlier. Firstly, how can domain-general widespread effects on neural processing lead, during the course of development, to the specific profile of autism in which some domains of thinking and sensation appear affected while others do not? Following this we will address the further issues of what brain mechanisms might underlie this variation from the typical developmental trajectory (Q. 2), why are specific brain regions associated with autism (Q. 3), and to what extent does the behavioral phenotype of autism reflect whole-brain adaptation at a particular developmental stage (Q. 4)?

## 4 Domain specific effects and uneven cognitive profiles (Question 1)

The causal factors underlying atypical neural processing can take a variety of different forms. For example, Rubenstein and Merzenich (2003) proposed that an atypical balance of excitatory and inhibitory activity within brain circuits may be a common feature of autism. While we have some understanding of the consequences of major imbalances between excitatory and inhibitory processes (e.g., epilepsy), the computational consequences of more mild imbalances or dysregulation in early development remain largely unknown. One of the functions of intrinsic inhibitory processing is to increase the signal to noise ratio by “cleaning up” spontaneous neural firing that is not directly linked to stimulus presentation or ongoing processing (Toyoizumi et al., 2013). However, on the flip side certain levels of background “neural noise” may in fact be critical to the development and specialization of cortical regions. Appropriate levels of noise can ensure that neural networks adaptively settle to appropriate configurations for the data processed, as it ensures that the network is not captured by local minima (Davis and Plaisted-Grant, 2015). However, excessive noise can also mask the appropriate signal, resulting in delayed opening of critical periods (Toyoizumi et al., 2013), delayed specialization of neural networks (Rubenstein and Merzenich, 2003) and changes in brain-wide connectivity (Eichler and Meier, 2008). These kinds of changes in the fidelity of neural processing could potentially occur for a number of reasons in addition to, or acting together with, genetic propensity.

The question of how such atypicalities can result in an uneven cognitive profile in which some skills can be near (or even better than) typical, while others show deficits has always posed a significant challenge to accounts of developmental disorders that postulate general factors, such as widespread synapse dysfunction, as being causal. It is commonly assumed that such widespread brain differences will necessarily have domain-general cognitive consequences and be accompanied by global delayed development. I believe this assumption to be incorrect, as it fails to take account of constructive and adaptive developmental processes (Karmiloff-Smith, 2009, Johnson, 2011). As discussed earlier, we have previously argued that the behaviors associated with autism can be interpreted as a natural adaptive developmental response to limited or sub-optimal neural processing early in postnatal life (see also Johnson et al., 2015 for details). In summary;

• Focal attention style – an adaptive response that restricts the quantity of information flow to help with parallel processing limits
• Repetitive behavior – self-generating predictable stimulation patterns that are easier to successfully compute than many real world events, particularly those in the social domain
• Withdrawal from social contexts – the most significant computational challenge an infant faces is the complex, multidimensional and dynamic stimulation associated with interpreting the behavior of others. Directing attention and processing resources to more comprehensible aspects of the early environment is more likely to maximize the fit between neural processing capacity and environment.

These are all examples of ontogenetic niche construction – an individual brain selecting those aspects of its environment which it is best suited to process, and generating behaviors to maximize sensory information that can be processed.

## 5 Adaptation through changes in network structure (Question 2)

The second question posed at the start of this paper is what are the neural mechanisms that underlie the adaptation of whole brain systems to adjust to differences in synaptic efficiency? In other words, how is progression along different developmental pathways instantiated in terms of human postnatal brain development? I will argue that this can occur through changes in both structural and functional brain networks.

Vértes and Bullmore (2014) characterize human brain development in terms of the increasing organization of structural and functional connectivity networks; a progression from near random networks to efficient “small world” networks (small world networks involve semi-independent “modules” containing many short-range connections being connected at a longer range to critical “hub” regions). Large-scale structural connections develop rapidly and achieve near-adult levels of network complexity within the first few years. During these first years there is scope for these developmental changes in structural connectivity to reflect aspects of the interaction between the brain and its environment. While functional connectivity also increases in complexity and organization during early years, the specificity of the mapping between structural and functional connectivity networks (i.e., the extent to which functional connectivity patterns are constrained by structural connectivity networks) appears to increase with age during development and up to adolescence (Hagmann et al., 2010). This observation is consistent with other reports of increasing constraints on structure-function relations with increasing age (Gordon et al., 2011) and closely related predictions from the Interactive Specialization framework (Johnson, 2011).

In terms of sensitive periods for human brain development, therefore, there are potentially two types of whole-brain network adjustment that could underpin ontogenetic adaptation. First, the construction of the structural connectivity network over the first two years may be open to influence by a variety of factors, including the previous ontogenetic history of brain functioning (Benders et al., 2015). Second, the less specialized network present in the infant brain allows for a broader mapping between the computations that underlie adaptive behaviors and their implementation across structural neural networks. In other words, during the first two years there may be several different options for how the computations necessary to support our species-typical behaviors can be implemented in terms of underlying structural connectivity. These options for implementing functional networks become narrowed to the most efficient configurations in the course of typical development (Vértes and Bullmore, 2014), along with their associated changes in underlying structural networks. However, initially other options are possible that may be evident in later life as different “styles” of neural processing. For example, a more featural and detail-oriented style of processing may reflect an alternate structure-function connectivity mapping for some key networks. Importantly, these adaptive changes may be difficult to reverse in later life even if there are changes in the external environment or internal neural processing.

How do these considerations relate to early perturbations to brain development, such as those discussed earlier? With early mild or focal damage, the majority of potential structure-function mappings may remain open to the individual for subsequent development, albeit with some delays or minor differences. With more widespread and prolonged perturbations to typical neural function early in development, the different options for structure-function mapping may become severely restricted, ending with mappings that are less optimal and/or result in different styles of processing. Moderate widespread perturbation may even change the profile of the most efficient options for subsequent network development, and these differences may become reflected in structural connectivity if they occur sufficiently early in postnatal development. In other words, the options are restricted to an alternative developmental route for structure-function mapping that brings both strengths and weaknesses in cognition and processing when compared to the typical profile.

An emerging literature is concerned with changes in network connectivity following acquired lesions in adults. Although the capacity for compensatory network responses in adults is likely to be reduced compared to the first 2 years of life, there are likely to be similarities in these changes. In general, following traumatic brain injury functional networks show a reduction in their “small world” architecture (Sharp et al., 2014) to a less differentiated state. More specific changes include a strengthening of connectivity to/from frontal regions, increases in the strength of the default mode network, and an apparent disconnection of network hubs (Fagerholm et al., 2015, Sharp et al., 2011).

Potential adaptive responses in network typology resulting from developmental damage are largely unknown. This issue has, however, been recently investigated for very preterm birth (Karolis et al., 2016). Structural and diffusion imaging data of the connectome associated with very preterm birth is associated with a strong “rich-club” architecture (rich club networks involve hubs that also tend to be interconnected with each other) in later life. While the precise computational implications of a strengthened rich club architecture are unclear, when these mediate between more modular networks this architecture can be efficient at both local and global levels (Markov et al., 2013). Thus, very preterm birth is associated with quantitatively stronger global structural connectivity, with rich club architecture receiving a disproportionate share of the more limited white matter resources. Karolis et al. (2016) suggest that these adaptive adjustments to network architecture may help ensure subsequent typical neurodevelopment, and that this structural network reorganization implies that certain regions will assume different roles within the overall architecture, i.e., there are different neurodevelopmental routes to a typical behavioral phenotype.

There is a mixed literature of findings on functional connectivity in autism with reports of both hypo- and hyper connectivity making it difficult to achieve consensus. Apparent inconsistencies have been attributed to developmental factors, methodological differences, or regional specificity. Most recently, Abbot et al. (2016) examined different core brain networks, including the Default-mode-network (DMN), and concluded that in autism there was generally less differentiation between activity networks. Specifically, reduced anti-correlations between typically segregated networks, and substantially different correlations between behavioral indices and network activity, implicates a whole-brain adjustment, rather than one confined to a specific region. Future research will need to ascertain the similarities and differences to the network adjustments associated with adaptive responses to more focal acquired damage.

Thus, a variety of adjustments to brain network typology are known to result from acquired lesions in adults or to very preterm birth. To some extent these changes reflect returns to earlier developmental stages (e.g., reduced “small worldness”). Whether the brain network changes observed in autism can be interpreted as an adaptation, and/or an alternative structure-function mapping, should be the topic of future research.

## 6 Specific brain region consequences of general neural atypicality (Questions 3)

Bringing together the first and second questions we have addressed, a third question arising is how a widespread neural atypicality induces apparently specific regional effects on the brain in autism? In many (or all) neuropsychiatric disorders, certain “hub” regions of the brain (regions with many connections) appear to be differentially affected (Crossley et al., 2014). For example, in autism critical hubs of the social brain network are often implicated (Pelphrey et al., 2014). This is potentially significant, as hub regions hold positions of functional importance through integrating information from different parts of the brain (Carter and Huettel, 2013). But hubs may also be particularly vulnerable (or at least show the clearest case-control differences) for several reasons. First, their higher metabolic rate may make them potentially more sensitive to pathogenic effects such as oxidative stress. Second, these regions are often the focus for convergence and integration of fine spatial and temporal resolution information, and are thus differentially sensitive to small changes in signal-to-noise-ratio, or slight E/I imbalances, that may have little effect on other regions. The importance of these hub regions for coordinating the activity of others may additionally make them harder to compensate for following damage, and therefore more likely to be implicated in clinical conditions. Thus, hubs combine both high topological value and high biological cost (Crossley et al., 2014). Therefore, when tracing causal pathways for neurodevelopmental disorders, it is important to note that apparently selective regional “deficits” in hubs could equally likely be a result of widespread and general atypicalities across brain tissue.

## 7 Plasticity, sensitive periods and alternative developmental trajectories (Question 4)

The final question that was raised in the Introduction concerned the extent to which the adaptive changes just described need to occur within a particular developmental sensitive period. I will argue that as a syndrome of adaptation a hallmark of later autism is that the process of brain adaptation is initiated within the first two (or three) years, even though the behavioral consequences of this may not become evident until later.

In the answers to Questions 1 and 2, it was concluded that adaptive structural connectivity changes over the first two years could constrain or change the subsequently available range of structure – function mappings in the brain. Alongside this process, self-generated changes in the postnatal environment experienced by the child helps it to compensate for its neural processing limitations, further reinforcing the neurodevelopmental changes to embed an alternative developmental pathway. However, some of the types of early neural disturbance associated with causes of autism, such as excitatory-inhibitory or neurotransmitter imbalances, could frequently be transient developmental glitches with a new homeostatic balance becoming restored in due course.

Although there is currently little direct evidence on this issue for autism, examples where transient adaptations have life-long consequences for brain development are common. For example, disruption to the GABAergic or glutamergic systems will likely have different effects in early development than in the mature brain. GABA is known to switch from a largely excitatory function in prenatal development toward inhibition in early postnatal development, and this switching may drive the opening and closing of critical periods in sensory cortices (Hensch, 2005, Hensch et al., 1998). Imbalances in excitatory and inhibitory activity are also likely to be significantly moderated by homeostatic cell mechanisms that maintain network stability in the mature brain (Turrigiano and Nelson, 2004, Turrigiano, 2011) in ways that vary across development. These complexities in developmental timing indicate that subtle disturbances in inhibitory/excitatory balance will vary across different phases of development. Finally, evidence from mouse models of neurodevelopmental disorders supports the proposal that some synaptic phenotypes are transient in nature (Kroon et al., 2013). For example, in fmr1 knock-out mice altered plasticity in somatosensory cortex that is observed during the first postnatal week becomes normalized by the third week (Harlow et al., 2010); this may relate to peak expression of the fmr1 gene, which is up-regulated between postnatal days 4 and 14 (Hoerder-Suabedissen et al., 2013). However, these earlier transitory atypicalities subsequently may have knock-on effects on later development stages (“sleeper effects”), which live on as secondary consequences of the initial imbalance. Thus, consideration of the role of disruptions of neurotransmitter systems in autism must take a developmental perspective.

Other aspects of human developmental biology show clear evidence for differential developmental pathways depending on early life experience. For example, a variety of related theories stem from “Barker’s hypothesis” (Barker and Osmond, 1986) in which he argued, based on an association between prenatal nutrition and late-onset coronary heart disease, that fetuses adapt to the environment that they expect to enter postnatally. According to this hypothesis the nutrient environment of the fetus usually also represents the expected postnatal environment. When the two match up all is well. However, when they do not, such as in a famine during fetal life followed by a postnatal period of plenty, the earlier adaptation can become harmful as the body is physiologically prepared for an environment that differs markedly from the one that it actually inhabits. Over ensuing years evidence for this view has accumulated, and the account updated to include peri and postnatal influences, and the notion of the “thrifty phenotype” in which fetal glucose conserving adaptation occurs in response to intrauterine hypoglycaemia, creating a significant mis-match when the postnatal nutritional environment is good, and thus increases the risk for several metabolic disorders, including type II diabetes (Hales and Barker, 2001). Similar hypotheses have been advanced for maternal stress during pregnancy and the resulting elevated glucocorticoid exposure of the fetus, resulting in long-term up regulation of the hypothalamo-pituatary-adrenal (HPA) axis after birth. Gluckman and Hanson (2004) classified these phenomena as “predictive adaptive responses”, that result in later disorders only when there is a mis-match between the predicted later environment and reality (for review see De Boo and Harding, 2006).

In an intriguing recent study, Filiano et al. (2016) have shown an association between immune system function, brain function and social behavior in mice. Reducing an immune system molecule, interferon gamma, is associated with overactive neurons in prefrontal cortex and poor social behavior. Restoring interferon gamma, returns prefrontal neuron activity to normal (suggesting increased inhibitory neuron activity), and appropriate social behavior. This association between the immune and nervous systems is hypothesized by the authors to be adaptive as infections spread more rapidly when animals are in close contact. Linking this to predictive adaptive responses is evidence that women who develop infections during pregnancy run an increased risk of having a child later diagnosed with autism (Brown et al., 2014; although other results are inconsistent – Zerbo et al., 2016). The intriguing association between prenatal maternal immune response, changes in brain function, and later social behavior, raises the speculative hypothesis that the adaptive brain trajectory we have discussed for autism, could at least partly reflect a predictive adaptive response for an environment rich in disease or environmental toxins.

## 8 Conclusions and future directions

In the paper I have addressed some key questions raised by a perspective on autism that views it as an alternative trajectory of human neural and behavioral development, and discussed how general factors may drive the alternative pathway to autism, and give rise to its uneven behavioral profile. I also discussed analyses of whole-brain connectivity that may underlie the adjustments in whole brain systems to accommodate early differences in synaptic processing efficiency. Finally, the issue of whether autism can result from only transient developmental disturbances, if these occur at critical points in early development, was addressed.

The views expressed herein stand in sharp contrast to widely held assumptions in child psychiatry. Autism is typically viewed from a “disease state” model in which its definition is frequently extended beyond the diagnostic behavioral phenotype, to an inferred lifelong brain or neurochemical pathology. However, we need to remember that the term “Autism” specifically refers to a diagnosis based on overt behavioral symptoms (at the end of a developmental pathway), and not to an identified underlying pathology. If the term is to be extended beyond its technical usage for a clinical diagnostic category, then I propose it should be taken to refer to a common alternative pathway of human brain development – the autism developmental pathway. Much effort in the field is currently being directed into stratifying the syndrome into “autisms”, each of which it is assumed will have its own different molecular and cellular pathways. In contrast, the approach outlined here suggests that this popular direction of research – again, primarily motivated by a static disease model – may turn out to be fruitless as there is a many-to-one mapping between the multiple potential causal factors and the unitary adaptive response. While there is no doubt some variation in the whole-brain adaptive response to early life brain atypicality, as it is an adaptive response there is no reason why the end state should precisely correspond to the original genetic or molecular deviations. By analogy, it’s impossible to tell from fever symptoms alone whether they are caused by a bacterial or a viral infection. Indeed, when studying the mechanisms of fever, the distal causal factors may not even be relevant to a satisfactory explanation of the phenomenon under study.

The perspective presented also suggests a number of new directions for future research that may be worth pursuing. While the notion of a unitary whole-brain adaptive response to a variety of different molecular, genetic and environmental factors discourages an overly reductionist approach to autism (see also, Johnson, 2015), it also discourages the current assumption that early infant predictive biomarkers will necessarily look like later emerging features of the diagnosis. We have previously argued that while there are clear emerging behavioral symptoms of autism evident in the second year, early predictive neurocognitive markers detectable during the first year often do not appear characteristic of these later symptoms (Johnson et al., 2015); hence, we have coined the term “antecedent” to refer to early predictors that appear unrelated in nature to the later diagnostic features. Indeed, in other cases of infant markers there may be a reversal of biomarker features between infancy and the later diagnosed condition, a hallmark of an adaptive process (Johnson et al., 2015).

Turning to genetic analyses of autism and related neurodevelopmental diagnoses such as ADHD, two general features appear to characterize investigations to date (Geschwind and Flint, 2015). First, the genetic aetiology of such conditions is complex with hundreds of different genes implicated, each in a small number of cases. Second, there is substantive overlap in these implicated genes between different neurodevelopmental conditions. From the perspective of the view advanced in this paper, these general observations are unsurprising in that we should expect a variety of genes associated with synaptic plasticity (adaptation) to be common across a variety of developmental conditions in which processes of adaptation or resilience are engaged. As discussed earlier, gene expression patterns between infants at-risk for autism and infants with acquired perinatal damage could be instructive.

Finally, it will be worth initiating MRI studies of structural and functional connectivity to assess the extent to which we see parallel changes to network topology resulting both from focal perinatal damage and from emerging autism, potentially indicating common mechanisms of adaptive plasticity becoming engaged.

## Conflict of interest

None.

## References

[bib0005] Abbot A.E., Nair A., Keown C.L., Datko M., Jahedi A., Fishman I., Muller R.-A. (2016). Patterns of atypical functional connectivity and behavioral links in autism differ between default, salience and executive networks. Cereb. Cortex.

[bib0010] Badawi N., Dixon G.S., Felix J.F., Keogh J.M., Petterson B., Stanley F.J., Kurinczuk J.J. (2006). Autism following a history of newborn encephalopathy: more than a coincidence?. Dev. Med. Child Neurol..

[bib0015] Barker D.J., Osmond C. (1986). Infant mortality, childhood nutrition, and ischaemic heart disease in England and Wales. Lancet.

[bib0020] Benders M.J., Palmu K., Menache C., Borradori-Tolsa C., Lazeyras F., Sizonenko S., Hüppi P.S. (2015). Early brain activity relates to subsequent brain growth in premature infants. Cereb. Cortex.

[bib0025] Bjorklund D.F. (2015). Developing adaptations. Dev. Rev..

[bib0030] Brown A.S., Sourander A., Hinkka-Yli-Salomäki S., McKeague I.W., Sundvall J., Surcel H.M. (2014). Elevated maternal C-reactive protein and autism in a national birth cohort. Mol. Psychiatry.

[bib0035] Carter R.M., Huettel S.A. (2013). A nexus model of the temporal–parietal junction. Trends Cognit. Sci..

[bib0040] Cicchetti D. (2013). Resilient functioning in maltreated children: past, present: and future perspectives. J. Child Psychol. Psychiatry.

[bib0045] Cicchetti D., Curtis W.J. (2007). Multilevel perspectives on pathways to resilient functioning. Dev. Psychopathol..

[bib0050] Crossley N.A., Mechelli A., Scott J., Carletti F., Fox P.T., McGuire P., Bullmore E.T. (2014). The hubs of the human connectome are generally implicated in the anatomy of brain disorders. Brain.

[bib0055] Davis G., Plaisted-Grant K. (2015). Low endogenous neural noise in autism. Autism.

[bib0060] De Boo H.A., Harding J.E. (2006). The developmental origins of adult disease (Barker) hypothesis. Aust. N. Z. J. Obstet. Gynaecol..

[bib0065] Edwards A.D. (2009). The discovery of hypothermic neural rescue therapy for perinatal hypoxic-ischemic encephalopathy. Semin. Pediatr. Neurol..

[bib0070] Fagerholm E.D., Hellyer P.J., Scott G., Leech R., Sharp D.J. (2015). Disconnection of network hubs and cognitive impairment after traumatic brain injury. Brain.

[bib0075] Eichler S.A., Meier J.C. (2008). EI balance and human diseases?from molecules to networking. Front. Mol. Neurosci..

[bib0080] Filiano A.J., Xu Y., Tustison N.J., Marsh R.L., Baker W., Smirnov I., Peerzade S.N. (2016). Unexpected role of interferon-γ in regulating neuronal connectivity and social behaviour. Nature.

[bib0085] Geschwind D.H., Flint J. (2015). Genetics and genomics of psychiatric disease. Science.

[bib0090] Gluckman P.D., Hanson M.A. (2004). Developmental origins of disease paradigm: a mechanistic and evolutionary perspective. Pediatr. Res..

[bib0095] Gordon E.M., Lee P.S., Maisog J.M., Foss-Feig J., Billington M.E., VanMeter J., Vaidya C.J. (2011). Strength of default mode resting-state connectivity relates to white matter integrity in children. Dev. Sci..

[bib0100] Hagmann P., Sporns O., Madan N., Cammoun L., Pienaar R., Wedeen V.J., Grant P.E. (2010). White matter maturation reshapes structural connectivity in the late developing human brain. Proc. Natl. Acad. Sci..

[bib0105] Hales C.N., Barker D.J. (2001). The thrifty phenotype hypothesis Type 2 diabetes. Br. Med. Bull..

[bib0110] Harlow E.G., Till S.M., Russell T.A., Wijetunge L.S., Kind P., Contractor A. (2010). Critical period plasticity is disrupted in the barrel cortex of FMR1 knockout mice. Neuron.

[bib0115] Hensch T.K. (2005). Critical period plasticity in local cortical circuits. Nat. Rev. Neurosci..

[bib0120] Hensch T.K., Fagiolini M., Mataga N., Stryker M.P., Baekkeskov S., Kash S.F. (1998). Local GABA circuit control of experience-dependent plasticity in developing visual cortex. Science.

[bib0125] Hoerder-Suabedissen A., Oeschger F.M., Krishnan M.L., Belgard T.G., Wang W.Z., Lee S., Molnár Z. (2013). Expression profiling of mouse subplate reveals a dynamic gene network and disease association with autism and schizophrenia. Proc. Natl. Acad. Sci..

[bib0130] Johnson M.H. (2011). Interactive Specialization: a domain-general framework for human functional brain development. Dev. Cognit. Neurosci..

[bib0135] Johnson M.H., Rutter M. (2015). Neurobiological perspectives on developmental psychopathology. Rutter’s Child and Adolescent Psychology.

[bib0140] Johnson M.H., Jones E.J., Gliga T. (2015). Brain adaptation and alternative developmental trajectories. Dev. Psychopathol..

[bib0145] Karmiloff-Smith A. (2009). Nativism versus neuroconstructivism: rethinking the study of developmental disorders. Dev. Psychol..

[bib0150] Karolis V.R., Froudist-Walsh S., Brittain P.J., Kroll J., Ball G., Edwards A.D., Nosarti C. (2016). Reinforcement of the brain’s rich-club architecture following early neurodevelopmental disruption caused by very preterm birth. Cereb. Cortex.

[bib0155] Kolevzon A., Gross R., Reichenberg A. (2007). Prenatal and perinatal risk factors for autism: a review and integration of findings. Arch. Pediatr. Adolesc. Med..

[bib0160] Kroon T., Sierksma M., Meredith R. (2013). Investigating mechanisms underlying neurodevelopmental phenotypes of autistic and intellectual disability disorders: a perspective. Front. Syst. Neurosci..

[bib0165] Low J.A., Galbraith R.S., Muir D.W., Killen H.L., Pater E.A., Karchmar E.J. (1988). Motor and cognitive deficits after intrapartum asphyxia in the mature fetus. Am. J. Obstet. Gynecol..

[bib0170] Markov N.T., Ercsey-Ravasz M., Van Essen D.C., Knoblauch K., Toroczkai Z., Kennedy H. (2013). Cortical high-density counterstream architectures. Science.

[bib0175] Marlow N., Rose A.S., Rands C.E., Draper E.S. (2005). Neuropsychological and educational problems at school age associated with neonatal encephalopathy. Arch. Dis. Child. Fetal Neonat. Ed..

[bib0180] Masten A.S. (2007). Resilience in developing systems: progress and promise as the fourth wave rises. Dev. Psychopathol..

[bib0185] Oliver A., Johnson M.H., Karmiloff-Smith A., Pennington B. (2000). Deviations in the emergence of representations: a neuroconstructivist framework for analysing developmental disorders. Dev. Sci..

[bib0190] Park H.J., Friston K. (2013). Structural and functional brain networks: from connections to cognition. Science.

[bib0195] Pelphrey K.A., Yang D.Y.J., McPartland J.C., Andersen S.L., Pine D.S. (2014). Building a social neuroscience of autism spectrum disorder. The Neurobiology of Childhood.

[bib0200] Reilly J.S., Bates E.A., Marchman V.A. (1998). Narrative discourse in children with early focal brain injury. Brain Lang..

[bib0205] Rubenstein J.L.R., Merzenich M.M. (2003). Model of autism: increased ratio of excitation/inhibition in key neural systems. Genes Brain Behav..

[bib0210] Sharp D.J., Beckmann C.F., Greenwood R., Kinnunen K.M., Bonnelle V., De Boissezon X., Leech R. (2011). Default mode network functional and structural connectivity after traumatic brain injury. Brain.

[bib0215] Sharp D.J., Scott G., Leech R. (2014). Network dysfunction after traumatic brain injury. Nat. Rev. Neurol..

[bib0220] Stiles J., Bates E.A., Thal D., Trauner D., Reilly J., Johnson M.H., Munakata Y., Gilmore R.O. (2002). Linguistic and spatial cognitive development in children with pre-and perinatal focal brain injury: a ten-year overview from the San Diego Longitudinal project. Brain Development and Cognition: A Reader.

[bib0225] Toyoizumi T., Miyamoto H., Yazaki-Sugiyama Y., Atapour N., Hensch T.K., Miller K.D. (2013). A theory of the transition to critical period plasticity: inhibition selectively suppresses spontaneous activity. Neuron.

[bib0230] Turrigiano G. (2011). Too many cooks? Intrinsic and synaptic homeostatic mechanisms in cortical circuit refinement. Ann. Rev. Neurosci..

[bib0235] Turrigiano G.G., Nelson S.B. (2004). Homeostatic plasticity in the developing nervous system. Nat. Rev. Neurosci..

[bib0240] Waddington C.H. (1966). Principles of Development and Differentiation.

[bib0245] Van Handel M., Swaab H., De Vries L.S., Jongmans M.J. (2007). Long-term cognitive and behavioral consequences of neonatal encephalopathy following perinatal asphyxia: a review. Eur. J. Pediatr..

[bib0250] Vértes P.E., Bullmore E.T. (2014). Growth connectomics: the organization and re-organization of brain networks during normal and abnormal development. J. Child Psychol. Psychiatry.

[bib0255] Volpe J.J. (2009). Brain injury in premature infants: a complex amalgam of destructive and developmental disturbances. Lancet Neurol..

[bib0260] Zerbo O., Traglia M., Yoshida C., Heuer L.S., Ashwood P., Delorenze G.N., Weiss L.A. (2016). Maternal mid-pregnancy C-reactive protein and risk of autism spectrum disorders: the early markers for autism study. Transl. Psychiatry.

